# Design and development of a biotreatment of *E. crassipes* for the decontamination of water with Chromium (VI)

**DOI:** 10.1038/s41598-021-88261-0

**Published:** 2021-04-29

**Authors:** Uriel Fernando Carreño Sayago

**Affiliations:** 1 Fundacion Universitaria los Libertadores, Facultad of Ingineering, Bogotá, Colombia; 2 Science Environmental and Sustainability of University Jorge Tadeo Lozano, Bogotá, Colombia

**Keywords:** Biotechnology, Environmental sciences, Engineering

## Abstract

The use of cellulose materials for the adsorption of heavy metals has increased in favorable results to comply with the removal of these contaminants from water, such as the case of Chromium (VI), being one of the most dangerous heavy metals for the environment and human health. The objective of this research is to design and develop a biotreatment with dry and crushed biomass of *E. crassipes* for the continuous treatment of Chromium (VI), determining through mathematical modeling the Fick diffusion constant (Kf), based on this constant Fick will establish the performance of the biotreatment and the intraparticle diffusion constant (Ks). The diffusion speed (Kf) of the biomass of *E. crassipes* chemisorbing Cr (VI) of 0.30 cm/min, also it got the constant of the adsorption capacities (Ks) was 0.0198 s. With (Kf) it can design the treatment systems according to caudal or load greatly contaminated, calibrating the parameters how caudal, volume, or area of contact of the system of treatment. Also with (Ks) will be possible the design and modeling of a treatment system to improve the capacity of adsorptions calibrating the density of the particle and the density of the contact bed of the treatment system. Based on Fick's second law, an equation was designed to determine the reliability and performance of water treatment systems through the *E. crassipes* plant.

## Introduction

Water is an increasingly deteriorated resource all over the world, due to corporate and government irresponsibility, where they have policies for its care, but very inefficient. Such is the case of the discriminated dumping of heavy metals into bodies of water, especially chromium, from the tanning industry, for example. This heavy metal in oxidation state (VI), it’s a carcinogenic compound, therefore, posing serious risks to human health^1,2^.

Recently, the removal of heavy metals by adsorption has found many applications due to its lower operating cost and more effectiveness than physical and chemical techniques. The operating cost of the adsorption technique can be drastically reduced with the use of cheaper or less expensive adsorbents^3^.

At present, different investigations in the world get specialized in finding economical and effective biotechnologies for the treatment of water contaminated with heavy metals. Adsorbent beds of heavy metals, such as chitosan^4^, coconut residues^5^, Banana^6^, bacterial cellulose^7,8^, nanocellulose^9,10^ and plant biomass^11–14^.

Vegetable cellulose is an interesting material due to its abundance and that this material is generally waste, like the biomass of *E. crassipes,* an abundant and practically wasted aquatic plant. Different investigations have demonstrated the power to purify waste and industrial waters^15–17^ due to the chemisorption process, a cation exchange process, between the functional groups of cellulose present in this plant (OH, NH_2_, COOH) and heavy metals^18–21^.

The development of treatment systems for adsorption columns (continuous system), with a fixed bed made up of biomass that adsorbs heavy metals, is a way to have disruptive projects because this type of treatment system provides a practical application in wastewater treatment^22–25^.

The adjustment and calibration of the continuous treatment systems could be coupled depending on the pollutant loads and the flow rate, simulating the performance in order to design ideal processes, using mathematical models^26,27^. A representative model is Fick's laws^28^ this law represents the chemisorption process of heavy metals in vegetable cellulose^29^.

The Fick diffusion model (Kf) has been used successfully to predict rupture curves, calibrate the inlet load and comply with the discharge standards, being the main parameter to model and define the designs of treatment systems^30–32^. The biomass adsorption capacities are modeled with the constant (Ks), it serves for the adjustment and calibration of the adsorbent bed together with the particle density^33,34^.

The objective of this research is the development of a biotreatment with dry and crushed biomass of *E. crassipes* for the continuous treatment of Chromium (VI), determining through mathematical modeling the Fick diffusion constant (Kf), and the intraparticle diffusion constant (Ks). Until now, in the open literature, obtaining the Fick diffusion constant (kf) and the intraparticle diffusion constant (Ks) in the chromium (VI) adsorption process by *E. crassipes* has not been obtained. Although the *E. crassipes* plant has been used for the adsorption of Chromium and lead in continuous processes, these design parameters of the treatment systems were not established^35^. Therefore, the novelty of this study is the implementation of a modeling approach to determine the constants (Kf) and (Ks). The result of this study will improve the design of the Cr (VI) adsorption process, creating an efficient and economical system to be carried out in industries.

## Materials and methods

A biotreatment with dry and crushed biomass of *E. crassipes* was developed for the continuous treatment of Cr (VI), determining through mathematical modeling the Fick diffusion constant (Kf), based on this Fick constant, the biotreatment performance will be established and the intraparticle diffusion constant (Ks).

**Figure 1:**
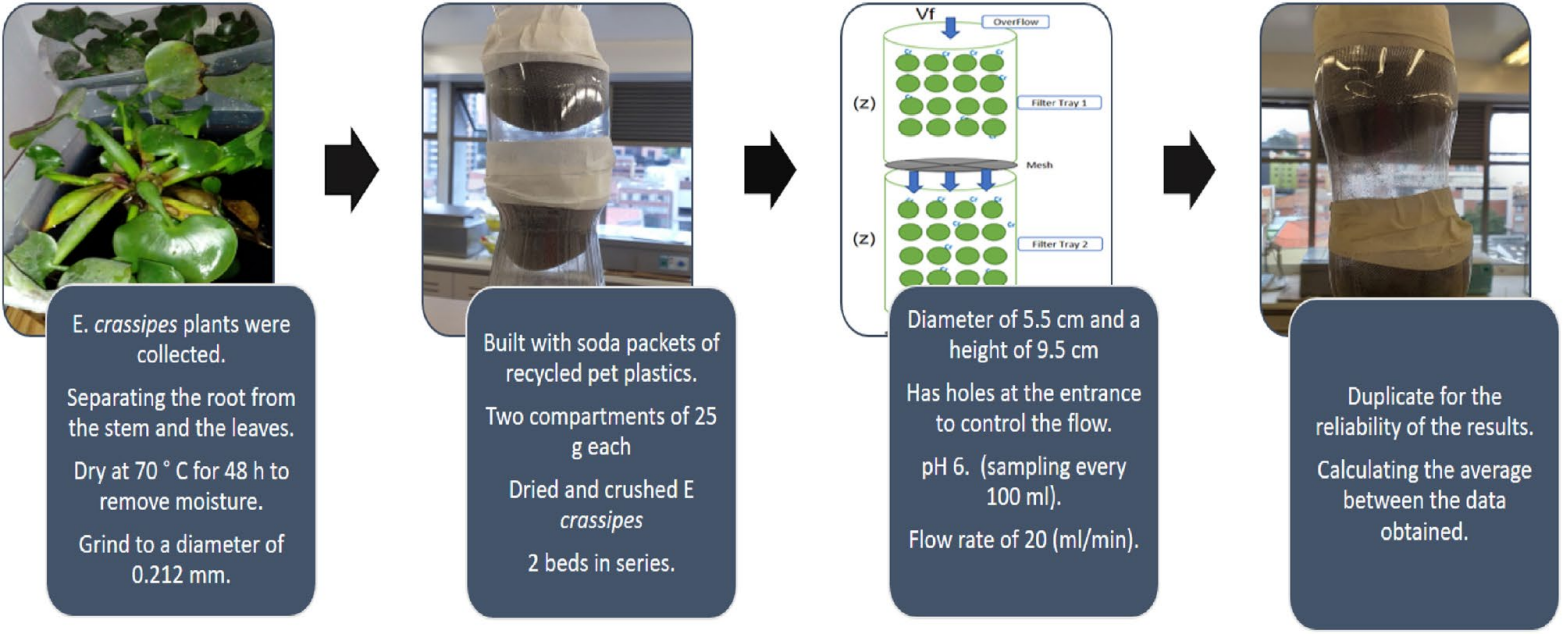
Process method.

The Fig. 1, show the process of built of biotreatment.

### Use of *E. crassipes* (CE)

According to decree-law 1376 (2013)^36^ of the Ministry of Environment and Sustainable Development of Colombia, the standard international by recollection wild sustainability of plants medicinal and aromatics (2006)^37^ and also the standard internationals Guidelines practice about the CITES and the medium of subsistence (2015)^38^, in the which regulates the scientific use of *E. crassipes* for environmental impact study for non-commercial, scientific research purposes, establishes that the maximum level of taxonomic detail must be taken from the plant to carry out the non-commercial, scientific study of environmental impact, the information associated with the collected specimens must also be characterized as: collection location (including altitude and geographical coordinates); collection date and collector.

The level of taxonomic is (*Eichhornia crassipes*). The collection location is the municipality of Mosquera, outside Bogotá DC, located at coordinates: 4.682995, − 74.256673, where the collection of a characteristic population of around 40 plants, already dead, according to with decree-law 2376 (2013) for projects experimental it is must take plants of all the wetland. Conducted on February 15, 2019, by researcher Uriel Fernando Carreño Sayago.

Then, washed (EC) with distilled water, separating the root from the stem and the leaves, conserving only the root of the plants. Then dry it at 70 °C for 48 h to remove moisture and grind to a diameter of 0.212 mm. The pulverized biomass will be sieved through a knife mill to obtain different sizes of particles.

### Chromium measurement

Spectrophotometer (Evolution 300 spectrophotometer) by monitoring changes in absorbance. All procedures for the determination of chromium, for water and substrates, were carried out by implementing APHA (Procedure of the American Public Health Association), for standard tests (standard methods for the examination of water and wastewater).

### Biotreatment experimentation

The biotreatments were built with soda packets of recycled pet plastics, the upper part was cut with scissors and joined to another soda packets to create two compartments of 25 g each of dried and crushed *E. crassipes* adsorbent material.

It has a fine nylon mesh, made of 2 beds in series, with a diameter of 5.5 cm and a height of 9.5 cm, it has holes at the entrance to control the flow, pH 6 The pH is equal to one of the tannery industry, (sampling every 100 ml), with a flow rate of 20 ml/min.

**Figure 2:**
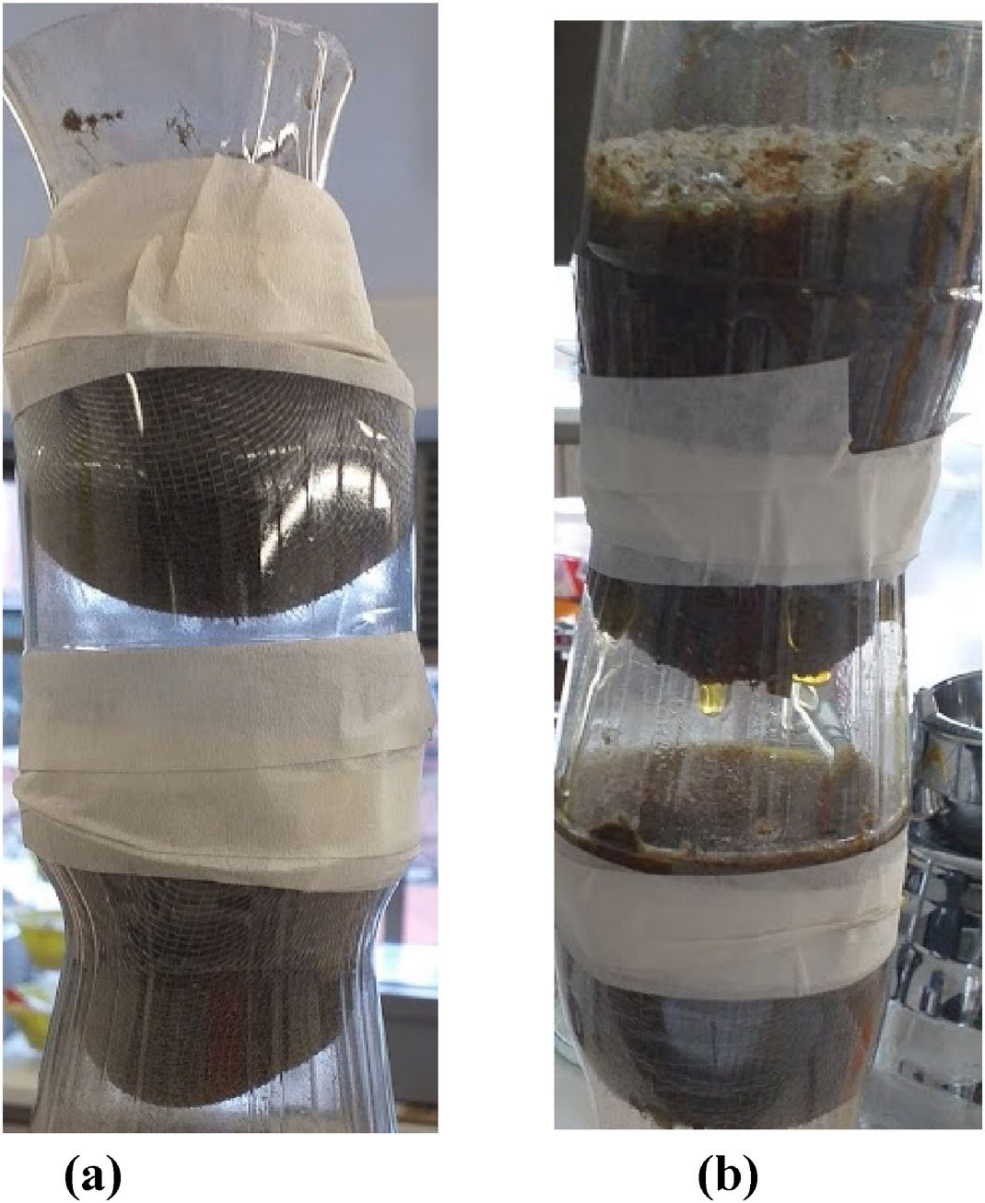
Biotreatment of Cr (VI) through biomass of *E. crassipes.*

Figure 2 shows two schematics of the experimental setup used, all the tests were carried twice for the reliability of the results, calculating the average between the data obtained with the percentage of metal removal. The sampling was carried out, nothing else reached the end of the flow, avoiding the chromium pressure.

### Chromium determination

Using the diphenylcarbazide method (the amount of chromium (VI) residue is estimated. For this purpose, the phosphate buffer solution was prepared by adjusting it to a pH equal to 2 with 90% of the grade of purity (H_3_PO_4_). In an eppendorf tube 200 µl of 0.5% diphenylcarbazide (with 97% the grade of purity) and (W/V acetone with 97 of grade of purity), 900 µl of phosphate buffer and 100 µl of the residual sample were added. A suitable portion is transferred to an absorption cell and the absorbance is measured at 540 nm.

### FTIR

The materials were characterized by Fourier Transform infrared spectroscopy (79 Jasco FTIR 430) to measure IR spectra in a spectral range of 4000–400 cm^−1^; a resolution of 4 cm^−1^, and a scanning speed of 2 mm s^−1^. And also by electron microscopy before and after the adsorption process.

## Results

It was realized characterizations of the biomass of *E. crassipes* through FTIR and BET.

### Analysis of FTIR

The spectra were measured on the Fourier transform spectrophotometer FTIR, *E.* *crassipes,* and *E.* *crassipes* with Cr (VI).

**Figure 3:**
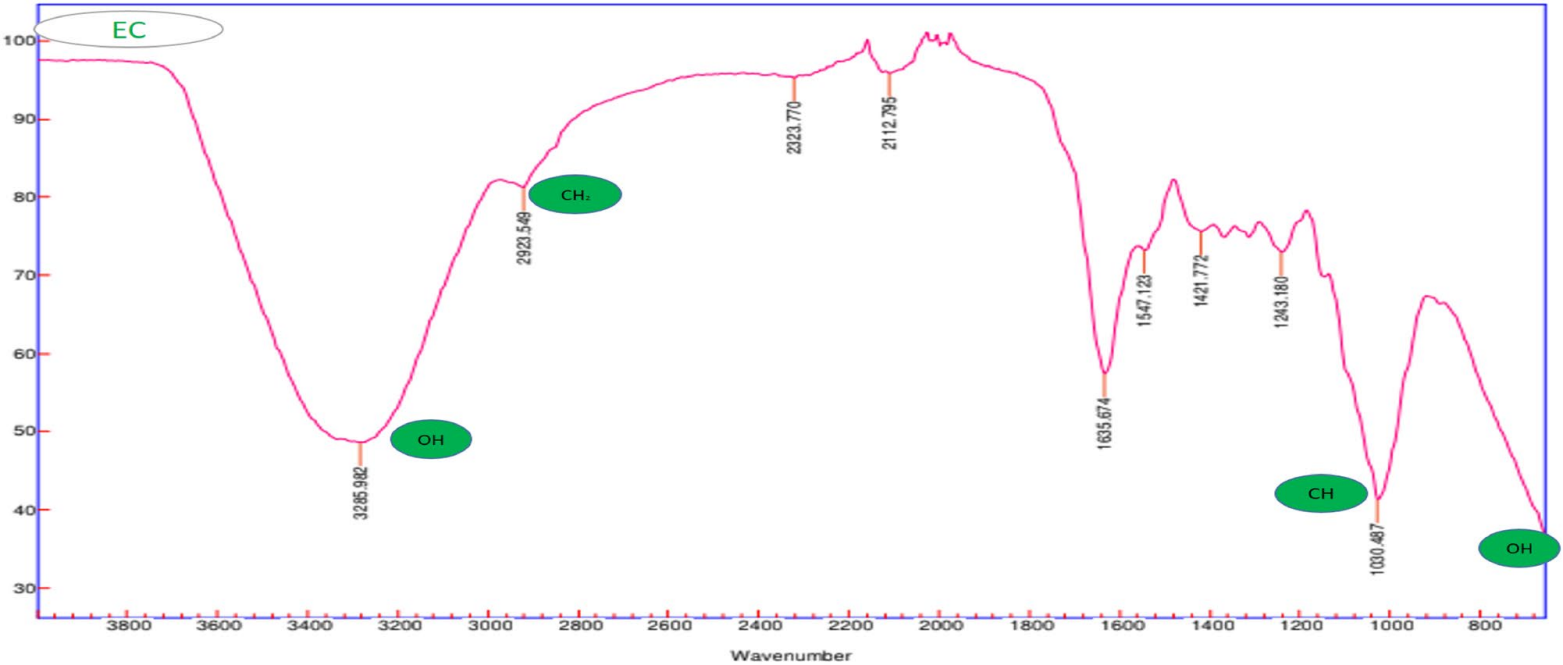
FTIR spectra of *E.* *crassipes* before Cr (VI) adsorption.

In Fig. 3, it shows the characteristic absorbance of *E.* *crassipes, *with a prolonged stretch in the hydroxyl group band (OH) in zone 3400, due to the existence of cellulose and lignin, and the stretching of the methyl group (CH_2_) can also be observed in the zone 2900 characteristic of plant biomass^39^ these groups are where heavy metals are hosted in biochemical adsorption processes, are tools for the interaction with these contaminants, especially the group (OH)^40^.

**Figure 4:**
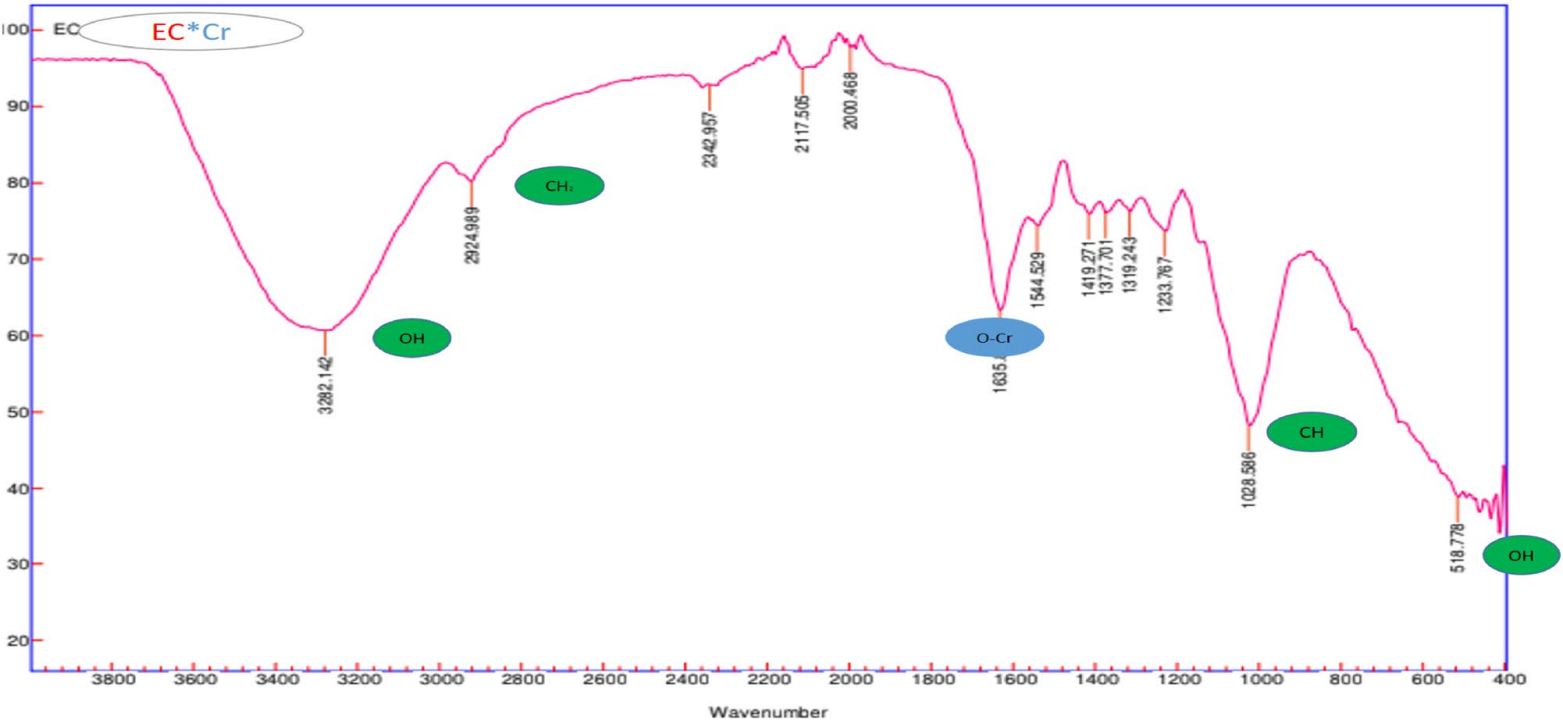
FTIR spectra of *E.* *crassipes* after Cr (VI) adsorption.

Figure 4 after adsorption of Cr (VI), indicating that *E.* *crassipes* adsorbed Cr (VI) as a function of the interaction with (OH), part of the (OH) groups were lost due to the formation of O–Cr ascension vibrations. This is represented in zone 1635, where it indicates the formation of carboxylic by the replacement of (H^+^) by Cr (VI) ions^41^.

### BET information (specific surface area; pore volumes, pore types)

The basic units of *E.* *crassipes* are characterized, porosity (ε), the density of the biomass (ρ), mass (ms), and the volume of the adsorbent (Vol).

1 $$\rho =\frac{ms}{Vol}.$$

The density particle (ρpart), this density is defined as the mass of the particle (mpart) over the small volume of the particle (Vpart) occupied in a space.

2 $$\rho part=\frac{mpart}{Vpart}.$$

The volume of each particle, assuming they are spherical, was calculated through the following Eq. (3).

3 $$Vpart= \frac{4\pi {rp}^{3}}{3},$$

where (r_p_) is the radius of the particle of sphere of *E.* *crassipes*, the relationship between densities is supported by the porosity design parameter (ε), this parameter relates the density of the particle with the density of the biomass used.

4 $$\varepsilon =1-\frac{\rho }{\rho part}.$$

According to Ref.^33^ the density of the particle must be higher than the density of the biomass, in order to guarantee the diffusion of the pollutant in the pores of the adsorbent material, concludes that, in treatment system designs, porosity values must be above 0.5. In Eq. (5), the surface area of the biomass used will be obtained.

5 $$As=\frac{3Vol(1-\varepsilon )}{rpart}.$$

The surface area is a function of 3 times the volume of the biomass occupied, together with the ratio of the densities of the particles used (ε), the surface area of the system is defined, dividing it on the density of the particle.

### Modeling development

The dynamics of the adsorption process will be reflected through Eq. (6),

6 $$m \frac{\partial q}{\partial t}=-v\frac{\partial c}{\partial t},$$

where (q) (mg/g), is the adsorption capacity of the biomass of *E.* *crassipes,* which is a function of time (t), (v) is the volume of the liquid to be treated, (m) is the quantity biomass used in the adsorption process and (c) is the concentration of chromium (VI) in liquid.

According to Ref.^33^ to establish this dynamism, the mass transfer equation for external diffusion or film will be used and can be derived from Fick's law,

7 $$Nf=Dl\frac{\partial C}{\partial \delta },$$

where: Dl is the diffusion coefficient in the aqueous phase (m^2^/s). (∂δ) is the biomass area of (*E. crassipes*) in the biotreatment, where adsorption takes place, (c) is the concentration of Chromium (VI) in liquid. The previous equation is integrated, obtaining a change in concentration over time and in the linked area of the process (δ).

8 $$Nf=Kf\left(co-c\right),$$

where (Kf) is the mass transfer coefficient of the film (m/t), (co) is the initial chromium concentration and (c) is the saturation concentration.

9 $$Kf=\frac{Dl}{\delta }.$$

According to Ref.^34^ the amount of chromium that has passed from the contaminated liquid to the biomass of (*E. crassipes*) per unit of time, *ṄF*, can be expressed through the material balance, relating Eqs. (6) and (8).

10 $$Nf=m \frac{\partial q}{\partial t}=-v\frac{\partial c}{\partial t}.$$

The derivatives indicate a variation of each variable with respect to time. The relationship between *ṄF* and the flow per unit area, *ṅF*, is given by: Eq. (11).

11 $$nf=\frac{Nf}{As},$$

where (*As*) is the total external surface of all the adsorbent particles. Equation (7) is divided into two different processes, m ∂q/∂t and v ∂c/∂t, since in both processes there are changes with respect to time, which are the concentrations (c) that decrease and the capacities of adsorption (q) that increase.

Taking the part of the changes in the concentrations of Eq. (6), drifting (∂c/∂t)

12 $$\frac{\partial c}{\partial t}=-kf\frac{As}{V}\left(co-c\right);$$

(Kf) is the transfer coefficient of the pollutant towards the *E. crassipes* particles, manifesting itself in the following way, (c0 − c). Through this equation, where the change in concentrations is a function of time.

It will be found through Eq. (12), where the change in concentrations is a function of time.

13 $$\frac{\partial c}{\left(co-c\right)}=-kf\frac{As}{Vl}dt.$$

Integrating Eq. (13), it remains:

14 $$\mathrm{Ln }\frac{C}{Co}=-kf\frac{As}{Vl}t.$$

Graphing this term, the logarithmic natural of concentration initial and concentration final, of Cr (VI), Ln C/Co Vs t, with the surface area (As) of contact together with the volume (v) in the experimental process of biotreatment, it will find the diffusion constant (kf) of Cr (VI) in the biomass of *E.* *crassipes.*

The calculation of ma ∂q/∂t of the adsorption capacity (q) is related to the density of the particle (pp), this is reflected by Eq. (15).

15 $$\frac{\partial q}{\partial t}=ppKs\left(qs-q\right).$$

(*ks*) is the internal mass transfer coefficient and (*qs* − *q̅*) is the difference of the adsorption capacities within the particle of (*E.* *crassipes*) and between the surface and the interior of the particle. To obtain the calculations of the chemical reactions perpetuated by the chemical diffusion process, Eqs. (12) and (15) are equated, leaving Eq. (16):

16 $$kspp\left(qs-q\right)=-kf\frac{As}{V}\left(c-cs\right),$$

where (*C*s) is the maximum concentration of Cr(VI) in (mg/L) in the liquid, and (*q*s) is the concentration of chromium inside E *crassipes,* practically its maximum capacity, the value of (*q*s) can be calculated using the following expressions, depending on the isotherm.

If it is Freundlich's Eq. (17):

17 $$qs=K{(Cs)}^{n}.$$

If it is Langmuir's Eq. (18):

18 $$qs=\frac{qmBCs}{1+B*Cs}.$$

In its linear form:

19 $$\frac{\mathrm{Cs}}{\mathrm{qs}}= \frac{1}{\mathrm{qm }}\mathrm{Cs}+\left(\frac{1}{\mathrm{qmB}}\right),$$

where, B is the Langmuir parameter.

If it is Temkin Eq. (20):

20 $$\mathrm{qs}=\left(\frac{\mathrm{R}}{\mathrm{B}}\right)\mathrm{Ln Kt}+\left(\frac{\mathrm{Rt}}{\mathrm{Bt}}\right)\mathrm{Ce},$$

21 $$bT=\left(\frac{\mathrm{R}}{\mathrm{B}}\right),$$

where: KT is the constant of the Temkin isotherm (L/mg); bT, constant related to the heat of adsorption; T, absolute temperature (K); R is the universal constant of gases (8.314 J/mol K) and B the constant related to the heat of adsorption (J/mol).

If it is Dubinin–Radushkevich isotherm Eq. (22):

22 $$\mathrm{Ln }\left(\mathrm{qs}\right)=\mathrm{Ln}\left(\mathrm{qm}\right)-\mathrm{B}{\varepsilon }^{2},$$

where *qm* is D–R isotherm constant representing the maximum adsorption capacity, *β* is the activity co-efficient related to mean free adsorption energy (mol^2^/kJ^2^) and *ε* is the Polanyi potential (kJ^2^/mol^2^) which is evaluated as:

23 $$\upvarepsilon =\mathrm{RT Ln}\left(1+ \frac{1}{Ce}\right),$$

where R is the universal gas constant (J/mol K) and T is the absolute temperature in K. Substituting the equilibrium equations of all the isotherms in the Eq. (16), the normalized general mass balance of these isotherms is defined as:

If it is Freundlich Eq. (24):

24 $$ks\times pp\times \left(Ks{\left(Cs\right)}^{n}-q\right)=-kf\frac{As}{V}\left(Co-Cs\right).$$

The resulting expression with Freundlich modeling, Eq. (25).

25 $$ks\times pp\times Ks{\left(Cs\right)}^{n}-Ks\times pp\times q=-kf\frac{As}{V}\left(C-Cs\right).$$

If it is Langmuir's Eq. (26):

26 $$ks\times pp\times \left(\frac{qmBCs}{1+B\times Cs}-q\right)=-kf\frac{As}{V}\left(Co-Cs\right).$$

The resulting expression with Langmuir modeling, Eq. (27).

27 $$kf\frac{As}{V}B{Cs}^{2}+\left(KsppqmB-qBKspp-Kf\frac{As}{V}CsB+Kf\right)Cs-qKspp-Kf\frac{As}{V}Cs=0.$$

If it is Temkin Eq. (28):

28 $$ks\times pp\times \left(\mathrm{bTLn Kt}+\mathrm{Ce }-q\right)=-kf\frac{As}{V}\left(c-cs\right).$$

The resulting expression with Temkin modeling, Eq. (29).

29 $$ks\times pp\times \mathrm{bT}\times \mathrm{Ln Kt}+ks\times pp\times \mathrm{Ce }-ks\times pp\times q=-kf\frac{As}{V}\left(c-cs\right).$$

If it is Dubinin–Radushkevich Eq. (30):

30 $$ks\times pp\times (\mathrm{qm}-{e}^{\mathrm{B}{\varepsilon }^{2}}) -q)=-kf\frac{As}{V}\left(c-cs\right).$$

The resulting expression with Dubinin–Radushkevich modeling, Eq. (31).

31 $$ks\times pp\times \mathrm{qm}-ks\times pp\times {e}^{\mathrm{B}{\varepsilon }^{2}} -q=-kf\frac{As}{V}\left(c-cs\right).$$

To determine the behavior of the adsorption reactions, it will be established through the experiments in biotreatment, which of these isotherms with their equations will be used.

**Figure 5:**
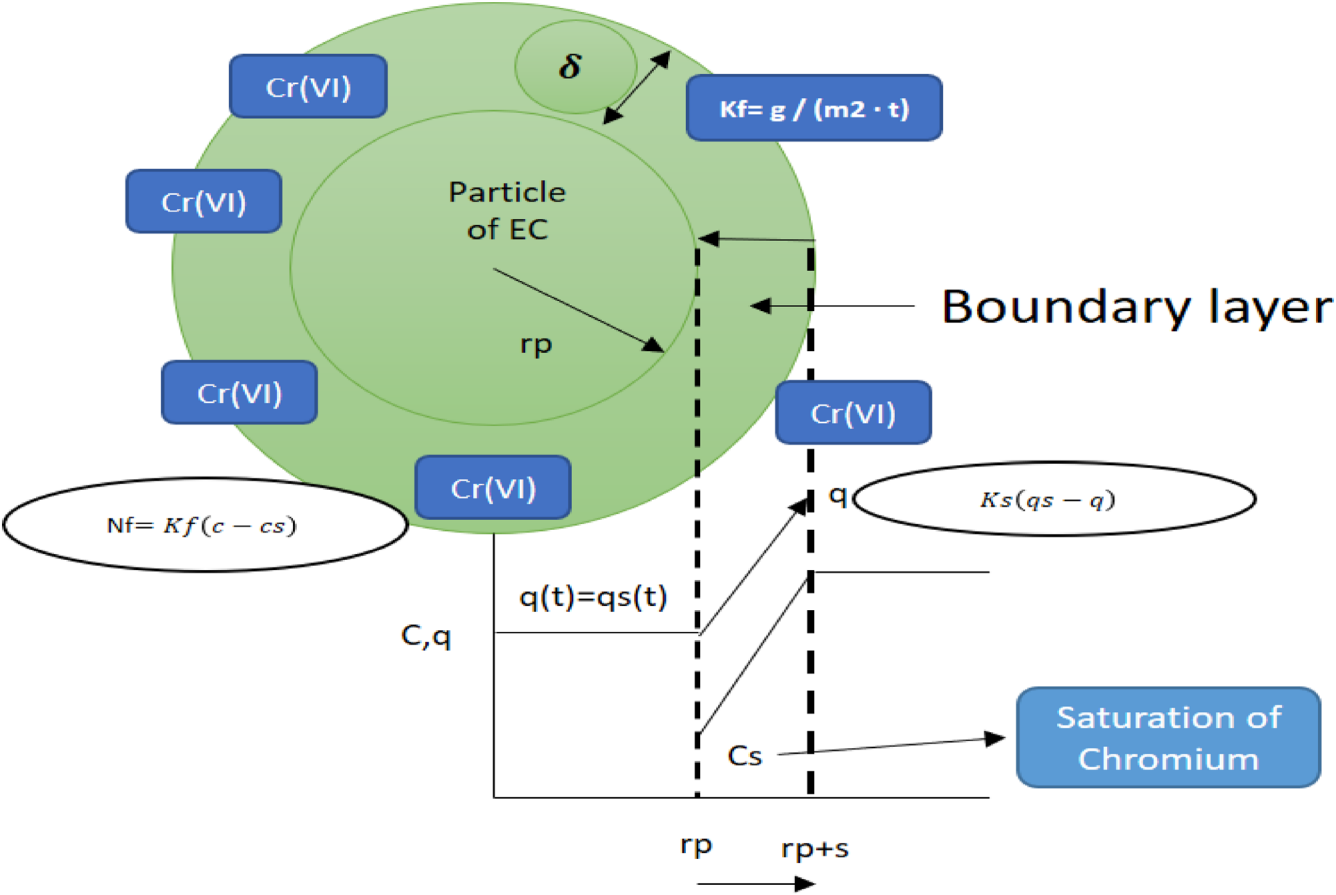
Representation of the *E.* *crassipes* sphere in the Chromium (VI) adsorption process.

In Fig. 5. it is specified where the chromium (VI) adsorption process occurs.

The constants (Kf) and (Ks) are one of the main objectives of this research since they relate to the amount of chromium pollutant transferred per unit of the established time. This value has not been determined in the literature, both for (Kf), transfer in the contaminant, and (Ks) transfer in the particle.

### Biomass yield measurement

With the defined diffusion constant (Kf), through Eq. (10), the second Fick's law will be related, obtaining the error of the adsorption capacities of the biomass of E *crassipes.*

32 $$\frac{Cs-Cx}{Cs-Co}=\left(1-\alpha \right)=\left(\frac{P}{\surd Kf\times T}\right),$$

where (Cs) is the saturation of Cr (VI) concentration, (Co) initial concentration, and (Cx) is the design concentration, at the time (x). The (p) is the depth given in cm, divided by the defined diffusion constant (kf) together with the time of the concentration variable (Cx). For the purposes of designing a treatment system, Fick's law on the adsorption capacities of biomass will be used.

33 $$Cs=Co-\frac{ma}{Vl}qb,$$

where, (qb) is the capacity reported in the Bath experimentation, together with the initial concentration (Co).

34 $$Cx=Co-\frac{ma}{Vl}qs,$$

where, (qs) is the capacity in continuous experimentation, together with the initial concentration. It is replaced in Eq. (34), with Eqs. (32) and (33)

35 $$\frac{Co-\frac{ma}{Vl}qb-Co+\frac{ma}{Vl}qs}{Co-\frac{ma}{Vl}qb-Co}=\left(1-\alpha \right)=\left(\frac{P}{\surd Kf\times T}\right),$$

36 $$\frac{-\frac{ma}{Vl}qb+\frac{ma}{Vl}qs}{\frac{ma}{Vl}qb}=\left(1-\alpha \right)=\left(\frac{P}{\surd Kf\times T}\right),$$

37 $$\frac{\frac{ma}{Vl}(qb-qs)}{\frac{ma}{Vl}qb}=\left(1-\alpha \right)=\left(\frac{P}{\surd Kf\times T}\right),$$

38 $$\frac{(qb-qs)}{qb}=\left(1-\alpha \right)=\left(\frac{P}{\surd Kf\times T}\right),$$

where, (qb): Capacity in batch (mg/g); (qc): Continuous capacity (mg/g); P: depth of the bed 30 cm; Kf: diffusion constant (cm/min) and T: rupture time. The error function α is represented by the representative adjustment to the possible errors in the experiment and will be complemented by the reliability that the system represents. The left part of the equation represents the error.

39 $$\frac{(qb-qs)}{qb}=\left(1-\alpha \right).$$

The concept of error underlies irrelevant random factors in the process^42^ perhaps external to the diffusion process of Cr (VI) adsorption by biomass. For this reason, the error concept of the equation of Fick's second law was adapted to establish the biomass yield of *E. crassipes.*

40 $$\mathrm{reliability}=1-\left(\alpha \right)=z.$$

The right part of Eq. (38) represents the standard z of the error, according to Fick's second law.

41 $$\mathrm{z}=\left(\frac{Pro}{\surd Kf\times T}\right).$$

This equation will be used to establish the reliability of the different capacities of biomass, under the different experiments of adsorption of heavy metals for part of the biomass.

### Experimental process

**Figure 6:**
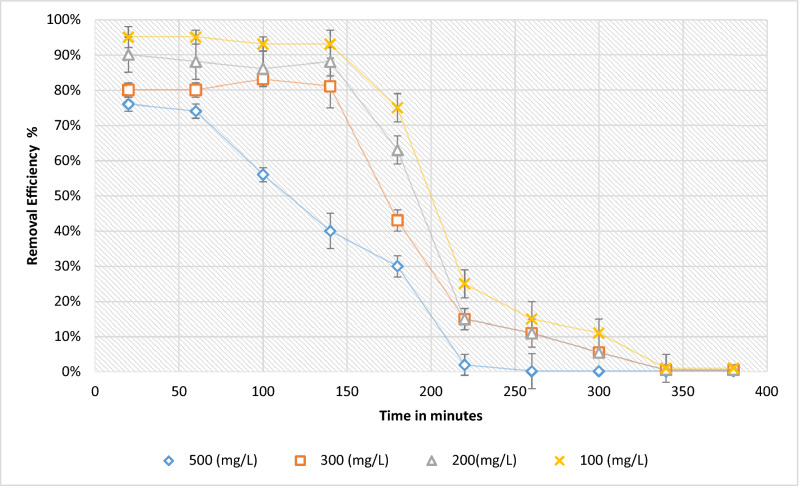
Percentages of removal of *E.* *crassipes.*

In the experimental process of biotreatment, is displayed the removals in Fig. 6, of the biomass treatment of *E.* *crassipes*, where are evidenced the percentages of remotions and the time of treatment; each point has the percentage of error, due to the all get the average of two experiments. It is appreciated that neither of the experiments was above 5% in the standards deviations.

In the initial removal processes with 500 mg/L, there were removals around 78% of Cr (VI), its breaking point being in the minute 60, around 900 mL of treated water. The yield with an initial concentration of 300 mg/L, obtained yields around 80%, its breaking point being at minute 140, treating around 1100 mL of water. For the treatment of 200 mg/L, treated around 1300 mL of water, with remotions of 90%. In initial concentrations of 100 mg/L, the biotreatment was 95% effective in the first sections, treating around 1400 mL in volume.

Achieved important results of 70% in removals of chromium and lead, with a smaller bed and with a longer height, treating these contaminates with *E.* *crassipes*^35^.

Used a packed bed column to treat Cr (VI) with the biomass of carbon synthesized, with the result interesting, removing around 80% of this pollutant, though the concentration initial was less than 50 mg/L^43^.

### Continuous adsorption isotherms

The adsorption isotherms were obtained in a continuous procedure to determine the diffusion behavior and determine the ideal equation by which the chromium chemisorption process from the biomass takes place.

**Figure 7:**
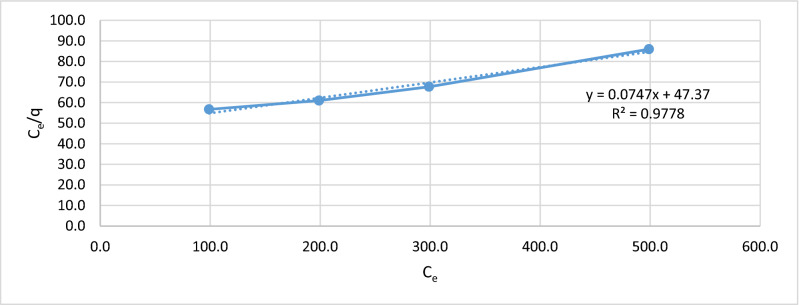
Langmuir isotherm.

**Figure 8:**
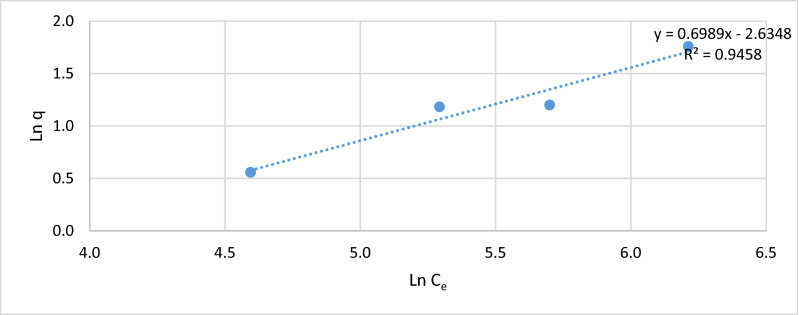
Freundlich isotherm.

Figures 5, 6, 7 and 8, show the behavior of the adsorption of biomass (*E.* *crassipes).* There were 4 different starting points, where the initial concentration of 100, 200, 300, and 500 mg/L of Cr (VI).

For the Langmuir isotherm, the graph of (C_e_/q) Vs C_e_ for Cr (VI) adsorption is shown in Fig. 7, representing the Langmuir equation.

An important adjustment can be observed, with an R2 of 0.9778, the adjustment is representative of this type of adsorption. Evidence of a monolayer adsorption process, through this equation, the maximum capacity of this biomass *E.* *crassipes* to retain metals was obtained, yielding the adsorption capacity of 5.7 mg/g in the continuous process of the filter. With Eq. (19), the Langmuir parameter B was obtained, which is 0.003.

To obtain the representations of the Freundlich isotherm, the (Ln) of the adsorption capacity (q) is obtained and the (Ln) of the final equilibrium concentrations (C_e_) is obtained, representing in Fig. 8.

**Figure 9:**
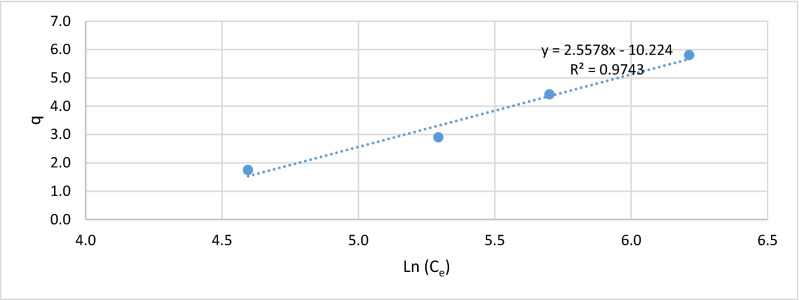
Temkin isotherm.

Also, it was revised the adjust of the isotherm Temkin, where through adsorption capacity (q) and the (Ln) the final equilibrium concentrations (C_e_) is obtained the parameters representing of this isotherm, representing in Fig. 9.

**Figure 10:**
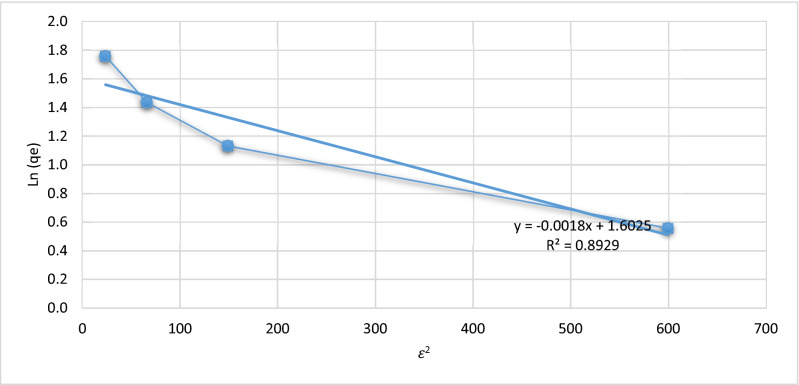
Dubinin–Radushkevich isotherm.

The isotherm Temkin is used in the process thermic^2^ due to this, it isotherm doesn’t represent the process adsorption of Cr (VI) for the *crassipes*. It was revised the adjust of the isotherm Dubinin–Radushkevich, where the of the (Ln) adsorption capacity (q) is obtained and the (ε^2^) is the Polanyi potential is obtained, representing in Fig. 10.

The Dubinin–Radushkevich isotherm establishes that the micropores of the adsorber are filled through a physical adsorption process^3^ due to this, this isotherm does not represent the adsorption process of *E.* *crassipes* towards Cr (VI). In the Table 1, shows to resume the parameters of each isotherm.

**Table 1:** Parameters each isotherms.

Isotherm	Constant	qm	R2
Langmuir	B = 0.03	7.9	0.977
Freundlinch	K = 0.69		0.94
Temkin	Kt = 10.8; Bt = 2.55		0.974
Dubinin–Radushkevich	B = 0.018	4.34	0.89

Through these graphs it can be established that biotreatment follows a behavior under the Langmuir isotherm, for this reason, the modeling equations of this isotherm will be used, therefore, Eq. (15) will be used to find the diffusion constant of the capacity Ks.

### Result BET information (pore volumes, pore types, and specific surface area)

The theory (BET) is an extension of the Langmuir isotherm^44,45^ and due to the adsorption adjustments of *E.* *crassipes* removing Cr (VI) with this isotherm, it was characterized under this theory.

The biomass used in the adsorption process will be 50 g, measuring the volume occupied by the amount of biomass used E *crassipes* yields 60 ml, with Eq. (1), the density of the biomass used in the present experiment was obtained.

$$\rho =\frac{50\mathrm{ g}}{80\mathrm{ mL}}=0.625\frac{\mathrm{g}}{{\mathrm{cm}}^{3}}.$$

The size of the diameter per particle for (EC) is 0.212 mm, similar observations have been reported^46^. With this value the volume of each particle was calculated, through Eq. (3).

$$Vpart= \frac{4\pi {\left(0.106\mathrm{ mm}\right)}^{3}}{3 }=0.005 {\mathrm{mm}}^{3}.$$

Taking the weight of the tiny particle of *E.* *crassipes*, with Eq. (2), the density of the particle is obtained:

$$\rho part=\frac{0.01(\mathrm{mg})}{0.005 (\mathrm{mm})}=2 \mathrm{mg}/{\mathrm{mm}}^{3}.$$

Through Eq. (4) the porosity of the particle of the biomass pores of *E.* *crassipes* will be established.

$$\varepsilon =1-\frac{0.625}{2}=0.68.$$

The relationship between the density of the particle and the biomass establishes an important relationship for the biomasses of *E.* *crassipes* due to their compact and important relationship in the pollutant diffusion process in this biomass, due to the porosity values above 0.5, in the designs of treatment processes, this relationship is decisive^33,34^^.^ This is achieved with a very finite particle diameter of less than 0.212 (mm).

With Eq. (5), the surface area used in the two biomasses was determined, in order to find the ideal contact of adsorbent and contaminant.

$$As\left(EC\right)=\frac{3\times 90\times \left(1-0.68\right)}{3.2}=36.7 {\mathrm{cm}}^{2}.$$

The surface area of 36.7 cm^2^ is the contact area between this biomass of *E.* *crassipes* and Cr (VI), which has a relatively smaller contact area with this contaminant. The external surface area has a strong influence on the rate of the mass transfer during the adsorption.

### Mathematical modeling

ith Eq. (14), it will be used to determine the diffusion constant of chromium (VI) in the biomasses of *E.* *crassipes.* By graphing this term Ln (C/Co) Vs t, the design parameters will be obtained, together with the diffusion constant of the chromium concentration on *E.* *crassipes.* Taking the value of (Kf), the constant (Ks) of Eq. (18) will be obtained.

**Figure 11:**
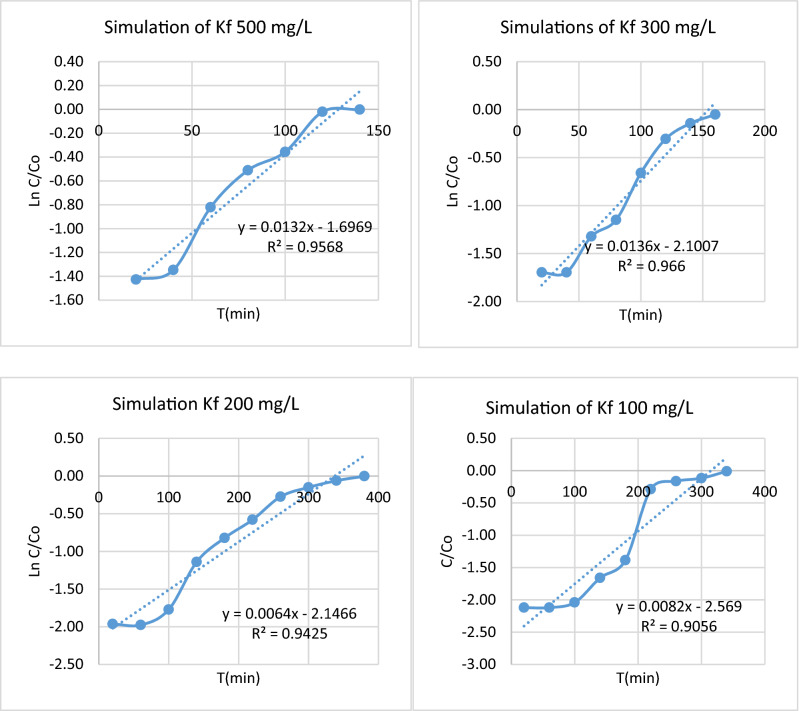
Simulation to find the diffusion constant Kf under different concentrations.

The resulting figurers are represented after the experimental process of *E.* *crassipes*, where each of the initial concentrations of 500, 300, 200, and 100 mg/L are shown. In the Fig. 11, show the simulation to find the diffusion constant Kf under different concentrations.

Through the previous graphs, the adsorption constants of the diffusion model were obtained. For example, in the adsorption experiment of initial 500 mg/L of Cr, Vs the adsorption time, the graph was obtained:

$$ y \, = \, 0.0132x \, {-} \, 1.69, \, R^{2} \, = \, 0.95 $$

Equation (9)

$$\mathrm{Ln}\frac{C}{Co}=-kf\frac{As}{Vl}t$$

Or

$$-kf\frac{As}{Vl}=0.013.$$

Through the equation was establishing that the filter area is 36.7 cm^2^ and the total volume of the process is 900 mL, yielding Kf = 0.32 cm/min, In the initial experiments of 300 mg/L of chromium, it showed 0.41 cm/min Kf in the graph, in the initial experiment 200 mg/L of chromium, it returned 0.23 cm/min and in the 100 mg/L initials yielded 0.31 cm/min. What could argue that the diffusivity of Cr (VI) in the biomass *E.* *crassipes,* has a constant Kf of 0.31 cm/min. In the Table 2, finds the constant of diffusion (kf).

**Table 2:** Constant of diffusion (Kf) (cm/min).

Experiments	Constant of diffusion (Kf) (cm/min)
500 (mg/L)	0.32
300 (mg/L)	0.41
200 (mg/L)	0.23
100 (mg/L)	0.31
Average	0.30
Standard deviations	0.06

The (Kf) of *E.* *crassipes* has a speed in the diffusion process (Kf) of 0.30 cm/min for chemically absorbing Cr (VI) as a constant, in experiments carried out by^34^, they found diffusion constants of 0.19 cm/min in biomass to retain phenols, similar to the present process.

For example, with the Eq. (14), having the Kf, of the *E.* *crassipes* biomass, the end concentration necessary to comply with some regulations could be adjusted, the input load to the system can also be adjusted, calibrating the design parameters that the treatment system would have, such as flow, volume and contact area.

### Relationship between biomass capacities

Through the second Fick's Law, the continuous yield of the biomass of *E.* *crassipes* will be established although Eq. (33):

$$\frac{(qb-qc)}{qb}=\alpha \left(\frac{P}{\sqrt{Kf}t}\right).$$

The adsorption capacity of (qb) is 7 (mg/g)^17^ on a batch laboratory scale, under the Langmuir isotherm model, it was found that the adsorption capacity (qb) in the filter in a continuous process is 5.7 mg/g. The diffusion constant (kf) is 0.31 cm/min, the depth is 36 cm, and the breakdown time is 200 min.

The relationship between the capacities $\frac{(qb-qc)}{qb}=\alpha$*,* reflect the relationship between the capacities found, both in batch and continuous, determining the probability of error (α), replacing values:

$$\frac{(7-5.7)}{7}=\left(1-\alpha \right)=\left(\frac{36}{\surd 0.31\times 400}\right)$$

$$\frac{(7-5.7)}{7}=0.18$$

$$\mathrm{reliability}=1-(\alpha )$$

$$\mathrm{reliability}=1-\left(0.18\right).$$

$$\mathrm{reliability}=0.82=82\mathrm{\%}.$$

$$\mathrm{Z}= 82\mathrm{\%}=\left(\frac{36}{\surd 0.31\times 900}\right).$$

The treatment process has a standard error of 0.18, being reliable its biomass treatment process of 82%, due to its total use. The standardized (z) relates the contact bed of the biomass with the pollutant, together with the removal constants (kf) and the contact time.

Solving Eq. (28),

$$\mathrm{Z}=\left(\frac{36}{\surd 0.31\times 900}\right)=0.22.$$

**Figure 12:**
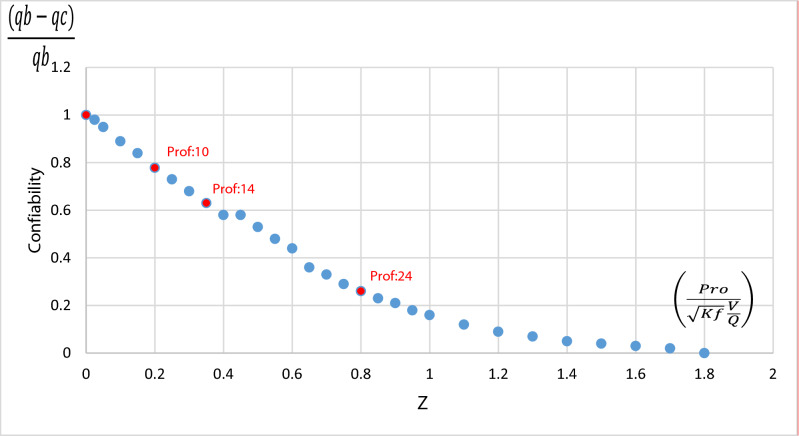
Reliability of the treatment system related to the standard z. (Adapted from Refs.^47,48^).

Looking at Fig. 12, it is observed that the process is 80% reliable.

If the contact surface were increased from 36 to 48 cm, according to Eq. (22), the biomass of *E.* *crassipes* would increase, decreasing the adsorption capacity continuously, affecting the reliability of the system, which would go from 80 to 60%. On the contrary, by reducing the contact area to 10 cm, the reliability increases to 85%.

Based on the second Fick equation, a reliability equation for biofilter treatment systems was found.

### Determination of the adsorption constant

With Eq. (27) the constant of the adsorption capacity (Ks) of the biomass of E *crassipes* will be determined when it is verified that the adsorption process had an adjustment to the Langmuir isotherm.

$$kf\frac{As}{Vl}B{Cs}^{2}+\left(KsppqmB-qBKspp-Kf\frac{As}{Vl}CB+Kf\right)Cs-qKspp-Kf\frac{As}{Vl}Cs=0$$

$$ks= \frac{kf\frac{As}{Vl}B{Cs}^{2}+Kf\frac{As}{Vl}Cs-KfCs+Kf\frac{As}{Vl}Cs}{ppqmCs-qBCspp-qpp},$$

where: Kf = 0.31 cm/min, As = 36 cm2, B = 0.03, Qm = 6.0, Cs = 100 mg/L, q = 5.7

Through this equation, the constant of the adsorption capacities (Ks) was 0.01, which indicates that at the rate of attraction Cr (VI) and retention, it structures from the biomass of *E.* *crassipes,* this value can be used in future research. In researches related to the adsorption process, they established the constant (Ks) in the retention of phenols by biomass, yielding 0.011 cm/min^34^.

Through Eq. (27), having (Ks) of biomass *E.* *crassipes*, it could be modeled and calibrated the design parameters of biotreatment such as the density of the particle and the density of the contact bed of this biomass.

## Conclusions

The biotreatment, based on a bed of dried and ground *E.* *crassipes,* had an efficient performance when removing Cr (VI), it is recommended for future research to use eluents that help optimize biomass.

The diffusion speed (kf) of the biomass of *E.* *crassipes* chemisorbing Cr (VI) of 0.31 cm/min was found, with this speed the development of treatment systems will be established for future research, based on the *E.* *crassipes* plant. The end concentration necessary to comply with some regulations could be adjusted, the input load to the system can also be adjusted, calibrating the design parameters that the treatment system would have, such as flow, volume, and contact area.

Based on Fick's second law, an equation was created to determine the reliability and performance of water treatment systems, where the decontamination process is through diffusion.

The constant of the adsorption capacities (Ks) was at 0.01 s, which indicates that at the rate of attraction Cr (VI) and retention, it in its structure from the biomass of *E.* *crassipes*, this value can be used in future research in the design of the system of treatment due to it can to adjust the density of particle and the density of the contact bed of this biomass, in order to improve the Cr (VI) adsorption capacities.
